# Giant Tunneling Electroresistance and Anisotropic Photoresponse in Sliding Ferroelectric Homojunctions Based on Bilayer Janus MoSSe

**DOI:** 10.3390/nano16060370

**Published:** 2026-03-18

**Authors:** Huxiao Yang, Yuehua Xu

**Affiliations:** School of Wang Zheng Microelectronics, Changzhou University, Changzhou 213164, China; s23060809030@smail.cczu.edu.cn

**Keywords:** two-dimensional materials, sliding ferroelectricity, electronic properties, tunneling electroresistance, optoelectronic properties

## Abstract

Interlayer-sliding ferroelectricity in van der Waals bilayers enables ultralow-power switching, but practical devices are often limited by contact/interface scattering and weak coupling between polarization and transport. We propose homophase lateral architectures based on bilayer Janus MoSSe: a 1T/2H/1T ferroelectric tunnel homojunction and an H-phase lateral p–i–n photodetector (artificially doped electrode). Metallic 1T electrodes largely eliminate contact barriers and maximize polarization-driven tunneling modulation. Using non-equilibrium Green’s function–density functional theory (Perdew–Burke–Ernzerhof approximation, without explicit spin–orbit coupling), we find that AB to BA sliding reduces the current from the nA range to the pA range, with the minimum current of|*I*_OFF_|_min_ = 2.83 pA, yielding giant tunneling electroresistance up to 5.3 × 10^4^%. Projected local density of states reveals a non-rigid long-range potential redistribution that reshapes the tunneling barrier and opens high-transmission channels. In the p–i–n photodetector, the response is strongly anisotropic and stacking-dependent: AB reaches photocurrent density *J*_ph_ ≈ 7.2 µA·mm^−2^ at 2.6 eV for in-plane light versus ≈ 2.9 µA·mm^−2^ at 3.5 eV for out-of-plane, and exceeds BA by 1.5–1.8 times due to density of states advantages and Mo-d orbital selection rules. Bilayer Janus MoSSe therefore provides a reconfigurable platform for high-contrast memory and polarization-sensitive photodetection.

## 1. Introduction

Ferroelectricity refers to the property of materials possessing spontaneous polarization that an external electric field can reversibly switch. Ferroelectric materials with low switching barriers are promising for meeting the dual demands of high-speed reading and low energy consumption in modern electronic devices [1]. Recently, sliding ferroelectricity has emerged in two-dimensional (2D) van der Waals (vdW) [2] layered materials as a novel physical mechanism for ultra-low-power polarization switching [3,4]. Specifically, this mechanism enables the switching of out-of-plane polarization via interlayer translation without accompanying significant lattice distortion or changes in chemical composition [5]. While single-layer vdW materials often lack ferroelectricity due to inversion symmetry, stacking them with interlayer displacement can break this symmetry and induce vertical polarization [6,7]. Theoretical and experimental works have demonstrated this phenomenon in various systems, such as h-BN [8,9,10], graphene [11,12,13,14], and transition metal dichalcogenide (TMD) bilayers [15,16,17]. Consequently, these 2D sliding ferroelectrics are considered to have broad prospects in applications ranging from electronic/optoelectronic memories [18,19] and photodetection [20,21,22] to neuromorphic computing [23,24].

Among the candidate systems, Janus MoSSe has attracted widespread attention due to its unique structural symmetry breaking [25,26,27,28]. By completely replacing S atoms on one side of a MoS_2_ monolayer with Se atoms, the asymmetric S/Se distribution induces an intrinsic out-of-plane dipole moment [29,30]. Furthermore, stacking two MoSSe layers introduces sliding ferroelectric degrees of freedom, allowing the system to switch between 2H semiconducting, 1T metallic, and distorted 1T’ phases [31,32,33,34]. However, despite the successful application of sliding ferroelectric bilayers in tunnel junctions [35,36,37,38], achieving high tunneling electroresistance (TER) remains a challenge. For instance, in In: SnSe/SnSe/Sb: SnSe lateral homojunctions, the zero-bias TER is reported to be 1.46 × 10^3^% [39].

In the realm of optoelectronics, MoSSe also exhibits unique advantages [40,41,42,43]. Previous studies have shown that its intrinsic out-of-plane dipole can generate a strong internal electric field, giving rise to the anomalous photovoltaic effect (APE) and pronounced photoresponse even without conventional p–n junctions [44]. Moreover, stacking engineering and graphene-based heterostructures have been explored to enhance the photocurrent density and response speed [45]. Nevertheless, these efforts predominantly rely on static stacking configurations or vertical dipoles [44,45]. In contrast, the dynamic reconfiguration of lateral carrier transport through switchable slip ferroelectric polarization in device-level p–i–n junctions remains largely unexplored in Janus MoSSe, especially for the purpose of achieving tunable and polarization-sensitive photodetection. In addition, existing device architectures often suffer from performance bottlenecks and limited functionality due to interface scattering and the weak coupling between polarization switching and transport states.

To address these issues, this study combines the advantages of sliding ferroelectricity with tailored device architectures. For electronic transport, we propose a lateral 1T/2H/1T homojunction, utilizing the metallic 1T-MoSSe phase as electrodes to potentially reduce the contact barrier and minimize interface scattering. We hypothesize that this pristine contact will maximize the modulation capability of sliding ferroelectricity, triggering a giant TER effect. In parallel, to investigate the optoelectronic functionality, we construct a lateral p-i-n junction based on the semiconducting H-phase bilayer, where the built-in electric field drives carrier separation. This design allows us to systematically explore how sliding polarization states regulate the photocurrent intensity and anisotropy.

In this work, we employ first-principles calculations combined with the non-equilibrium Green’s function (NEGF) method to systematically investigate the transport and optoelectronic properties of bilayer Janus MoSSe. This ferroelectric switching dramatically reconstructs the tunneling barrier in the 1T/2H/1T homojunction, leading to a giant TER up to the order of 10^4^%. Furthermore, in the H-phase p-i-n photodetector, we find that the device exhibits strong optical anisotropy and polarization-dependent photocurrents, where the AB state outperforms the BA state, and in-plane light polarization induces a much stronger response than out-of-plane polarization. This study provides a theoretical basis for designing low-power non-volatile memories and tunable 2D optoelectronic devices based on sliding ferroelectricity.

## 2. Materials and Methods

### 2.1. Geometry Optimization and Electronic Structure Calculation Methods for Bilayer MoSSe

Bilayer MoSSe structural optimization and electronic property calculations were performed using the QuantumATK (S-2021.06, Synopsys Inc., Sunnyvale, CA, USA) package based on DFT [46,47,48]. The calculations employed the Perdew–Burke–Ernzerhof (PBE) method within the generalized gradient approximation (GGA) and the linear combination of atomic orbitals (LCAO) method [49]. The SG15 pseudopotentials were adopted to replace the all-electron potentials, and the wave functions were expanded using a high-accuracy numerical basis set [50]. The real-space density mesh cutoff was set to 75 Hartree. A 16 × 16 × 1 k-point grid was used to sample the first Brillouin zone of bilayer MoSSe. The convergence criterion for structural optimization was set to the maximum force on each atom, which was less than 0.001 eV/Å. To accurately account for the vdW interactions at the interface, the Grimme DFT-D2 semi-empirical correction was applied, and a vacuum spacing of 25 Å was introduced to avoid interlayer interactions. The energy barrier along the ferroelectric switching pathway was evaluated using the climbing-image nudged elastic band (CI-NEB) method, with the maximum force on atoms set to 0.05 eV/Å [51]. The spontaneous polarization of different bilayer MoSSe configurations was calculated using the Berry-phase method [52].

DFT calculations are carried out within the scalar-relativistic approximation, i.e., without explicitly including SOC in the self-consistent electronic structure. Previous first-principles studies on Janus MoSSe have shown that SOC mainly introduces valley-dependent spin splittings near band edges and slightly renormalizes the band gap, while leaving the semiconducting character and the orbital nature of the band-edge states essentially unchanged. For example, Cheng et al. pointed out that whether or not SOC was present, MoSSe maintained a direct band gap. SOC mainly caused a spin splitting of approximately 0.1–0.2 eV in the valence band at the K point. In this case, MoSSe exhibited only a small Rashba spin splitting, without qualitatively altering the band gap properties or the characteristics of the band-edge states [53]. Long et al. pointed out that the effect of SOC is mainly confined to the vicinity of the K point, while the dispersion and the position of the band edges near the Γ point remain largely unchanged [54]. Since the key analysis of this work primarily focuses on the band-edge physics near the Γ point, SOC does not have a significant impact on our results.

### 2.2. Calculational Method of the I–V Characteristics Curve

The electronic transport calculations were performed by combining the NEGF formalism with DFT. The PBE functional and the LCAO approach were adopted, and SG15 pseudopotentials were used for the carrier transport calculations. For the *I*–*V* characteristic calculations, the Brillouin-zone k-point grid was set to 8 × 1 × 84. The *I*–*V* characteristics of the two-probe configuration were evaluated based on the Landauer–Büttiker equation, as follows [55]:

(1) $$I\left(V_{bias}\right)=\frac{2e}{h}\int T\left(E,\varepsilon_{L},\varepsilon_{R}\right)\times \left[f_{R}\left(E,\varepsilon_{R}\right)-f_{L}\left(E,\varepsilon_{L}\right)\right]dE$$

where ${\;V}_{bias}\;$ denotes the bias voltage applied between the two electrodes $eV_{bias}=\varepsilon_{R}-\varepsilon_{L}$, *T* is the carrier transmission coefficient, and $f_{L}(E,\varepsilon_{L})$ and $f_{R}(E,\varepsilon_{R})$ correspond to the Fermi–Dirac distribution functions of the left and right electrodes, respectively.

### 2.3. Calculational Method of Photocurrent

The photocurrent density was calculated within the framework of first-order perturbation theory, based on the first Born approximation, and combined with the NEGF approach [56,57]. The perturbation induced by the electron–photon interaction is defined by the Hamiltonian as follows:

(2) $$\hat{H}={\hat{H}}_{0}+\frac{e}{m_{0}}A\cdot \hat{p}$$

where ${\hat{H}}_{0}$ is the Hamiltonian of the two-probe system, $m_{0}$ is the free-electron mass, $\hat{p}$ denotes the electron momentum operator, and A is the electromagnetic vector potential. The transmission coefficient is calculated as follows [58]:

(3) $$T_{\alpha }\left(E\right)=Tr\left\{i\Gamma_{\alpha }\left[\left(1-f_{\alpha }\right)G_{ph}^{<}+f_{\alpha }G_{ph}^{>}\right]\right\}$$

where $G_{ph}^{<}$ and $G_{ph}^{>}$ denote the lesser and greater Green’s functions based on the Keldysh equation, $\Gamma_{\alpha }$ and $f_{\alpha }$ correspond to the linewidth (broadening) function and the Fermi–Dirac distribution function of the α (left or right) electrode, respectively. The photocurrent density *J*_ph_ is calculated as follows [59]:

(4) $$J_{ph}=\frac{e}{S\hbar }\int \frac{dE}{2\pi }\sum_{\alpha }T_{\alpha }\left(E\right)$$

where e is the electron charge, S is the cross-sectional area of the material, and ℏ is the reduced Planck’s constant. The integral term represents the summation over the energy E, accounting for the contribution of electron transmission at different energy states.

In addition, the exchange correlation functional, basis set, and pseudopotentials used in the photocurrent calculations were the same as those employed in the electronic transport calculations.

## 3. Results and Discussion

### 3.1. Geometric Structure and Electronic Properties of Bilayer MoSSe

**Figure 1:**
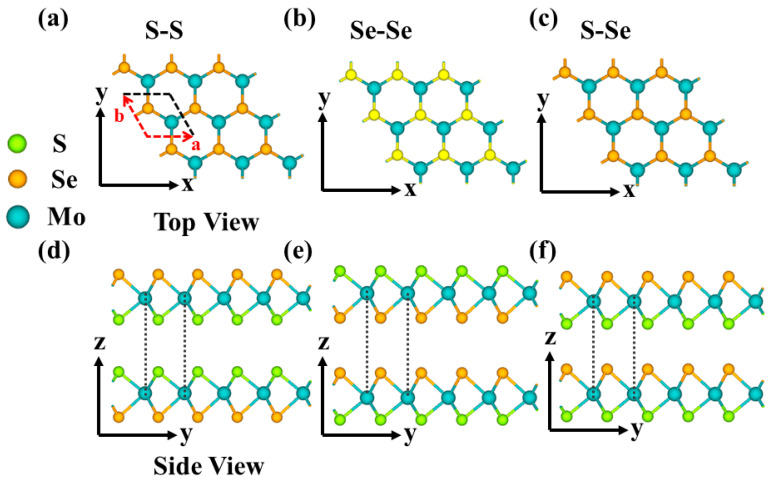
(**a**–**c**) Top views of the AA-stacked bilayer MoSSe in three different configurations: S–S, Se–Se, and S–Se; (**d**–**f**) Side views of the AA-stacked bilayer MoSSe in the same three configurations.

First, this study starts with an analysis of the geometric structure and electronic properties of bilayer MoSSe. MX_2_-type TMDs are vdW layered materials composed of X–M–X sheets, in which the transition-metal (M) layer is sandwiched between two chalcogen (X) layers. MoSSe is a Janus structure with an X–M–Y configuration, formed by substituting one S layer in MoS_2_ with Se atoms. As shown in Figure 1, three bilayer MoSSe stacking configurations can be constructed according to the sequence of atomic-layer arrangement, namely S–S, Se–Se, and S–Se. All three configurations adopt AA stacking, where the Mo atoms in the upper and lower layers are perfectly aligned.

**Figure 2:**
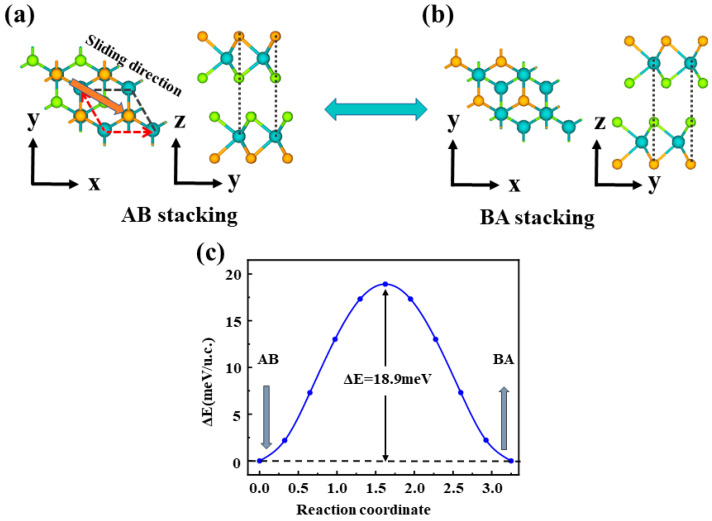
Schematic illustration of the transition between two FE phases of the S–S configuration (**a**) top and side views of the AB-stacked structure, The red arrow represents the sliding direction used to switch polarization; (**b**) top and side views of the BA-stacked structure; (**c**) the transition energy barrier along the ferroelectric switching pathway. The switching pathways for the Se–Se and S–Se configurations are similar to those of the S–S configuration, as shown in Figures S1 and S2.

Owing to the weak interlayer interaction, vdW structures can change their stacking configurations through interlayer sliding, thereby breaking the original symmetry and generating configurations with distinct electronic properties. To explore the impact of symmetry breaking on the electronic characteristics, it is necessary to further investigate how different stacking configurations affect the polarization states and the associated electronic properties. Figure 2 shows two ferroelectric (FE) phases of the S–S configuration. Starting from AA stacking as the parent structure, one MoSSe layer is laterally slid to form AB or BA stacking. In the AB stacking, the Se and S atoms in the upper layer are located directly above the Mo atoms in the lower layer, whereas in the BA stacking, the Mo atoms in the upper layer are located directly above the Se and S atoms in the lower layer. The AB and BA stackings can be switched by a relative lateral translation along the vector $\vec{r}$= ($\vec{a}$ + $\vec{b}$)/3, which changes the interlayer coupling and consequently modifies the electronic properties of the two stackings.

The minimum energy path for ferroelectric switching in bilayer MoSSe was obtained using the CI-NEB method. For the S–S stacking, the transition from the AB to the BA configuration needs to overcome an energy barrier of 18.9 meV/u.c., as shown in Figure 2c, suggesting that polarization switching via interlayer sliding is energetically accessible. For the Se–Se and S–Se stackings, the switching barriers are 21.2 meV/u.c. and 22.4 meV/u.c., respectively (Figures S1 and S2). The similarly small barriers indicate that sliding-mediated polarization reversal is intrinsically favorable across all three stacking configurations. This result is generally consistent with previous reports on the low-energy polarization reversal behavior of Janus MoSSe [34].

### 3.2. Giant Tunneling Electroresistance in Lateral Ferroelectric Homojunctions

**Figure 3:**
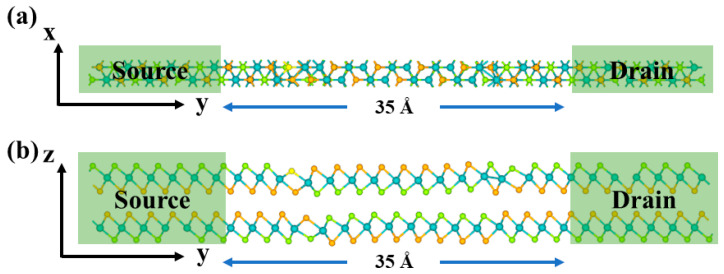
Schematic structure of the 1T-MoSSe/2H-MoSSe/1T-MoSSe sliding ferroelectric tunnel homojunction based on bilayer MoSSe: (**a**) top view and (**b**) side view. The length of the central tunneling region is 35 Å.

After verifying the stable sliding ferroelectricity, we construct a 1T/2H/1T lateral homojunction (Figure 3) to investigate the transport properties. The intermediate 2H-phase bilayer MoSSe serves as the ferroelectric tunneling layer and the central scattering region, while the 1T-phase MoSSe at both ends serves as homojunction metallic electrodes. On the one hand, this homophase design can significantly reduce interfacial strain and band mismatch, thereby suppressing interface scattering. On the other hand, it provides a sufficient density of metallic states, which facilitates the amplification of the modulation effect arising from the sliding ferroelectric polarization states in bilayer MoSSe at the device scale. The transport direction of the device is defined along the y-axis within the layer (Figure 3b).

**Figure 4:**
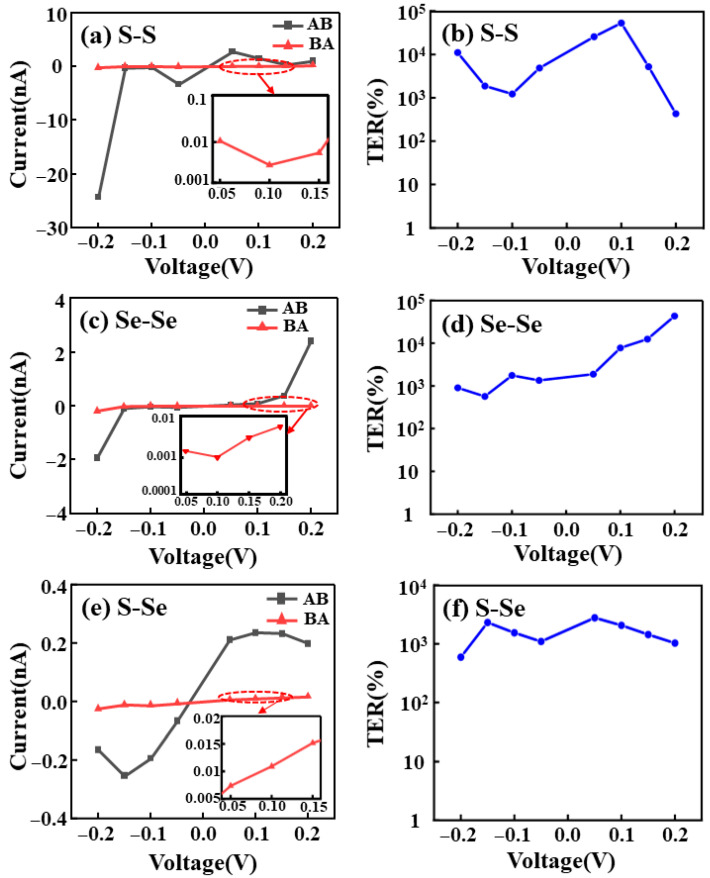
(**a**) I–V characteristic curves of devices with AB and BA stacking in the S–S configuration; (**b**) TER for the S–S configuration; (**c**) I–V characteristic curves of devices with AB and BA stacking in the Se–Se configuration; (**d**) TER for the Se–Se configuration; (**e**) I–V characteristic curves of devices with AB and BA stacking in the S–Se configuration; (**f**) TER for the S–Se configuration.

Figure 4 presents the *I*–*V* characteristics and tunneling TER for different configurations. When the OFF-state current approaches zero, the TER would diverge in theory. However, in our NEGF-DFT calculations, the OFF-state current remains finite and varies smoothly with bias. Since the current sign simply reflects the transport direction under opposite bias polarities, the magnitude (|*I*|) is used when assessing the OFF-state level. In our dataset, the minimum OFF-state current magnitude is |*I*_OFF_|_min_ = 0.00283 nA (2.83 pA), clearly nonzero, indicating that the large TER arises from genuine suppression of tunneling current rather than a spurious divergence associated with a vanishing denominator or numerical underflow. Moreover, the calculated current levels (from pA in the OFF state to nA in the ON state) fall within the detection capability of low-current transport measurements, indicating that the predicted TER readout is experimentally accessible [60]. For the S–S configuration (Figure 4a), the device in the AB polarization state exhibits conductive behavior under negative bias, with the current rapidly increasing to the order of tens of nA. In contrast, the BA state remains highly insulating. This indicates a strong dependence of the transport properties on the sliding polarization stacking. Accordingly, the TER is calculated as follows [61]:

(5) $$\mathrm{TER}=\frac{I_{ON}-I_{OFF}}{I_{OFF}}\times 100\%$$

where *I*_ON_ and *I*_OFF_ are obtained at the same bias from the AB (ON) and BA (OFF) polarization states of the same 1T/2H/1T homojunction.

This results in a giant TER on the order of 10^3^% to 10^4^% across the observable bias window (Figure 4b) with a maximum of 5.3 × 10^4^% under our definition. This performance represents a significant breakthrough compared to existing designs reported in the literature. For instance, the zero-bias TER is reported to be 1.46 × 10^3^% in In: SnSe/SnSe/Sb: SnSe homojunctions [39]. Furthermore, this high-performance switching is universal across different stacking types. For the Se-Se configuration (Figure 4c,d), the TER peaks at nearly 10^4^% at 0.2 V bias. Similarly, for the S-Se configuration (Figure 4e,f), the AB state current reaches 0.3 nA, and the TER consistently exceeds 10^3^%, approaching 10^4^%. Table 1 summarizes the TER values of this work and compares them with those in previous studies, highlighting the improvement achieved in this study.

These results further demonstrate that reversing the sliding ferroelectric polarization state can universally realize high-contrast tunneling electroresistance modulation in the three configurations. Overall, the AB/BA sliding polarization stackings of bilayer MoSSe not only constitute a pair of stable and switchable ferroelectric polarization states, but also can markedly alter the ON/OFF states of the device in the lateral tunnel junction, giving rise to a strong polarization-dependent and high-TER effect.

**Figure 5:**
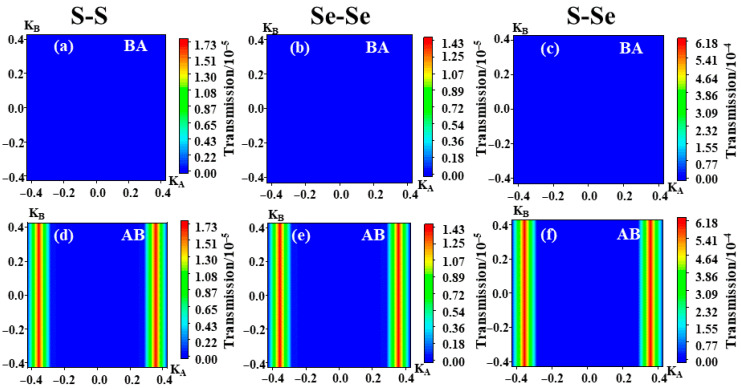
k_||_-resolved transmission probability at the Fermi level for the bilayer MoSSe sliding ferroelectric tunnel homojunction (1T-MoSSe/2H-MoSSe/1T-MoSSe), corresponding to the BA and AB stackings: (**a**) BA stacking in the S–S configuration; (**b**) BA stacking in the Se–Se configuration; (**c**) BA stacking in the S–Se configuration; (**d**) AB stacking in the S–S configuration; (**e**) AB stacking in the Se–Se configuration; (**f**) AB stacking in the S–Se configuration.

To uncover the quantum origin of this giant TER, we calculate the momentum-resolved transmission at the Fermi level (Figure 5). It should be noted that, to maximize the impact of polarization switching on the k_||_-resolved transmission spectrum, the k_||_-resolved transmission spectrum shown here is calculated under the applied bias voltage that yields the largest TER. In the BA state, transmission is suppressed across the entire Brillouin zone. Conversely, in the AB state, we observe high-transmission “hot spots” near k_A_ ≈ ±0.35, indicating the opening of high-transmission channels. These high-transmission channels significantly enhance the total transmission coefficient near the Fermi level, thereby resulting in the conductance of the AB polarization state being significantly higher than that of the BA state. This striking contrast in the k_||_-resolved transmission spectra provides an intuitive momentum-space picture of the substantial difference in the number of available transport channels between the two sliding ferroelectric polarization states. It thereby provides strong corroboration for the subsequent real-space barrier analysis based on the Projected Local Density of States (PLDOS).

**Figure 6:**
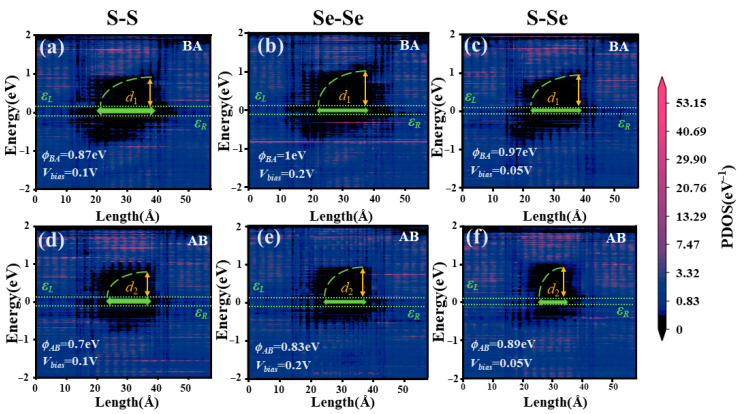
PLDOS of the 1T-MoSSe/2H-MoSSe/1T-MoSSe junction for different stackings: (**a**) BA stacking in the S–S configuration; (**d**) AB stacking in the S–S configuration; (**b**) BA stacking in the Se–Se configuration; (**e**) AB stacking in the Se–Se configuration; (**c**) BA stacking in the S–Se configuration; (**f**) AB stacking in the S–Se configuration.

We further analyze the PLDOS along the transport direction y (Figure 6) to reveal the barrier remodeling mechanism. It should be noted that the PLDOS distributions are all calculated under the applied bias voltage at which the TER reaches its maximum for the corresponding configuration. These results, obtained at the bias giving the largest TER, illustrate the effect of polarization switching on the local density of states and the effective potential barrier. The 1T-MoSSe electrodes (y = 0–11 Å and 46–57 Å) maintain stable metallic states, while the central tunneling region (y = 11–46 Å) dictates the transport. For the S-S configuration in the BA state (Figure 6a), the conduction band forms a high and wide potential barrier. The barrier height is approximately 0.87 eV, and the effective width spans d_1_ ≈ 14 Å (y = 24–38 Å). In the AB state (Figure 6d), the polarization flip shifts the conduction band downward. This not only lowers the barrier height to 0.7 eV but also significantly shrinks the effective width to d_2_ ≈ 10 Å (y = 26–36 Å). A similar trend is observed for Se-Se configuration (Figure 6b,e), where the barrier height decreases from 1 eV (BA) to 0.83 eV (AB), and the width contracts from d_1_ ≈ 14 Å (y = 22–36 Å) to d_2_ ≈ 11 Å (y = 25–36 Å). The S–Se configuration (Figure 6c,f) also exhibits the same trend, with the BA state being “high and wide” and the AB state “low and narrow.”

In the BA polarization state, electrons must traverse an effective barrier region that is both high and wide, leading to a substantially reduced tunneling transmission probability and thus a larger tunneling resistance. In contrast, in the AB polarization state, the barrier height decreases, and the effective width is reduced, allowing electrons to pass through the barrier region more readily, yielding a higher transmission probability and a lower tunneling resistance. This trend is fully consistent for all three configurations, indicating that sliding ferroelectric polarization reversal effectively regulates the lateral tunneling current by reconstructing the LDOS and reshaping the effective barrier profile. This constitutes the essential mechanism underlying the giant TER in Janus MoSSe sliding ferroelectric tunnel homojunctions. Figure 6 quantitatively reveals the polarization dependence of the effective barrier morphology in real space. However, to further address the causal origin of the TER on the order of 10^4^% at the mechanistic level, more direct evidence is still required to show that polarization reversal first alters the long-range self-consistent electrostatic environment inside the tunneling region, with barrier reshaping and the opening/closing of transport channels emerging as natural consequences.

**Figure 7:**
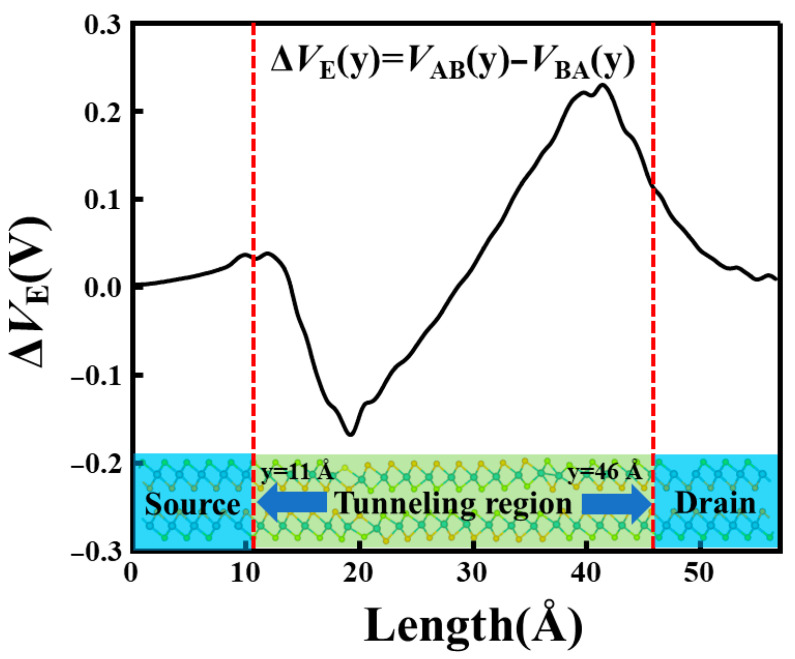
Difference in the macroscopic-averaged electrostatic potential between the AB and BA polarization states. The dashed lines mark the boundaries of the tunneling region at y = 11 Å and y = 46 Å. The bipolar profile indicates the reversal of interface bound charges driven by the sliding ferroelectricity.

The physical driver for this synergistic barrier remodeling is the long-range electrostatic reconfiguration, as evidenced by the macroscopic average potential difference $∆V_{E}(y)\;=\;V_{\mathrm{AB}}(y)-V_{\mathrm{BA}}(y)$ in Figure 7. Δ*V*_E_ exhibits a distinct bipolar feature in the tunneling region: a negative valley of −0.17 V near 20 Å at the source interface and a positive peak of +0.23 V near 40–42 Å at the drain interface, resulting in a substantial potential variation of ~0.4 V. The physical origin of the bipolar $∆V_{E}(y)\;$ profile originates from the reversal of ferroelectric polarization and the associated reversal of interfacial bound charges. Across a polar discontinuity, the bound charge density is given by σ_b_ = P⋅$\hat{n}$. Therefore, switching from AB to BA stacking reverses P_z_ and flips the sign of σ_b_ at each 1T/2H interface, leading to opposite local electrostatic potential steps in the two polarization states. Consequently, the interface where *V*_AB_ is lower than *V*_BA_ appears as a negative extremum in Δ*V*_E_, while the opposite interface becomes a positive extremum. Because the finite ferroelectric region must satisfy overall electrostatic neutrality, the bound charges and fringing fields at the two ends have opposite signs, enforcing an antisymmetric (bipolar) Δ*V*_E_ distribution. T This polarization-driven bipolar potential redistribution shifts the local band edges in opposite directions at the two interfaces, thereby reshaping the tunneling barrier and enhancing the TER behavior. Such a potential reconfiguration is directly manifested in the PLDOS in Figure 6. Meanwhile, the accompanying changes in band alignment and interfacial coupling conditions provide the necessary conditions for the formation of the high-transmission stripes in the AB polarization state in Figure 5.

### 3.3. Photocurrent Transport Properties

**Figure 8:**
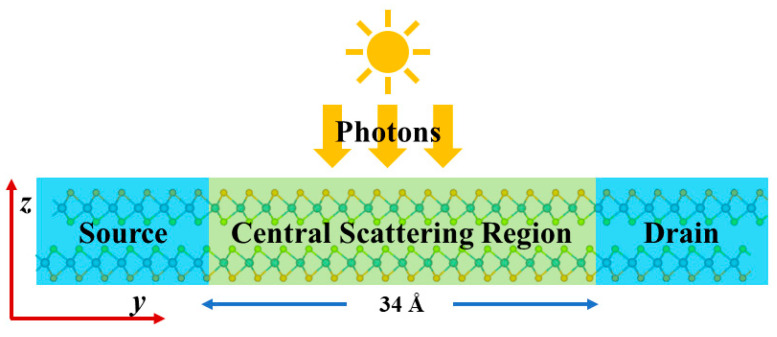
Schematic structure of the bilayer MoSSe p–i–n junction photocurrent device.

In addition to electronic transport, polarization reversal can also have an important impact on the optoelectronic properties. To investigate how sliding polarization modulates the photocurrent response, we construct a bilayer MoSSe p–i–n junction optoelectronic device model (Figure 8). The device consists of a source, a drain, and a central scattering region, with the central scattering region having a length of approximately 34 Å. The source and drain serve as the carrier injection and collection terminals, respectively, while the central scattering region is the primary area for light absorption and photogenerated carrier generation. Under p–i–n doping, the left end is p-doped (−0.01 e/atom) and the right end is n-doped (+0.01 e/atom), with an intrinsic middle region. When the device is illuminated, incident photons are absorbed in the central scattering region to generate electron–hole pairs. Driven by the built-in electric field or an applied bias, the photogenerated electrons and holes migrate toward the drain and source, respectively, producing an observable photocurrent.

In the photocurrent calculations, the PBE functional was employed. This approach is expected to yield reliable results for the heavily doped p–i–n structure. In p–i–n structures, the built-in electric field dominates carrier separation, and PBE can accurately capture the essential transport characteristics that determine device performance [64]. Meanwhile, the enhanced screening effects and weakened electron–hole interactions caused by high carrier concentrations also reduce the influence of many-body effects [65,66], which has been validated in two-dimensional photodetectors such as MoS_2_ and WSe_2_ [67,68,69]. Therefore, PBE can still provide reliable predictions for the trends in the calculated photocurrent. As shown in Figure 8, the device transport direction is defined as the y-axis parallel to the MoSSe layer plane, while the direction perpendicular to the layer plane is defined as the z-axis. Therefore, the z- and y-polarizations discussed in the text correspond to the out-of-plane electric field and the in-plane electric field (along the transport direction), respectively.

**Figure 9:**
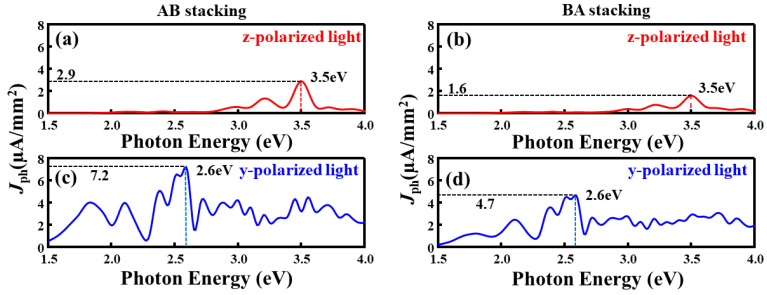
Photocurrent device based on the S–S configuration: (**a**) photocurrent response of the AB stacking under z-polarized illumination; (**b**) photocurrent response of the BA stacking under z-polarized illumination; (**c**) photocurrent response of the AB stacking under y-polarized illumination; (**d**) photocurrent response of the BA stacking under y-polarized illumination.

Having elucidated the transport modulation mechanism, we further investigate the impact of sliding ferroelectricity on the optoelectronic performance. Figure 9 presents the photocurrent spectra of the S-S configuration under different stacking orders and light polarization directions. The device exhibits a strong dependence on both the ferroelectric polarization state and the optical polarization angle. When the incident light is polarized along the out-of-plane direction (z-polarized), the photocurrents for both AB and BA stackings exhibit main peaks at a photon energy of approximately 3.5 eV, which falls in the near-ultraviolet (UV) region, indicating a pronounced UV photoresponse. The near-UV peak is scientifically informative because it accesses higher-energy interband excitations and offers an additional spectral handle to quantify the stacking- and polarization-dependent photoresponse [70]. However, their intensities differ markedly: the peak photocurrent density for the AB stacking reaches *J*_ph_ ≈ 2.9 μA·mm^−2^, which is approximately 1.8 times that of the BA stacking (1.6 μA·mm^−2^). Notably, when the light polarization is switched to the in-plane direction (y-polarized), two significant changes are observed: a redshift of the main peak to 2.6 eV, and a drastic intensity surge. The peak photocurrent density for the AB stacking jumps to *J*_ph_ ≈ 7.2 μA·mm^−2^, while the BA stacking also increases to 4.7 μA·mm^−2^. These peak photocurrent densities are even better than those of other 2D materials. For example NaCuTe (1.6 μA·mm^−2^) [71], NaCuSe (0.65 μA·mm^−2^) [71], and InSe (0.018 μA·mm^−2^) [72]. Overall, the AB stacking consistently maintains a performance advantage (approx. 1.5 times higher than BA), and the in-plane response is significantly stronger than the out-of-plane response, demonstrating pronounced optical anisotropy. This trend is universal; for instance, in the Se-Se configuration, the z-polarized photocurrent peaks at 3.55 eV (AB) and 2.75 eV (BA), whereas the y-polarized response shifts to lower energies with peaks at 2.45 eV and 2.9 eV (AB), further confirming the robust tunability of the system.

**Figure 10:**
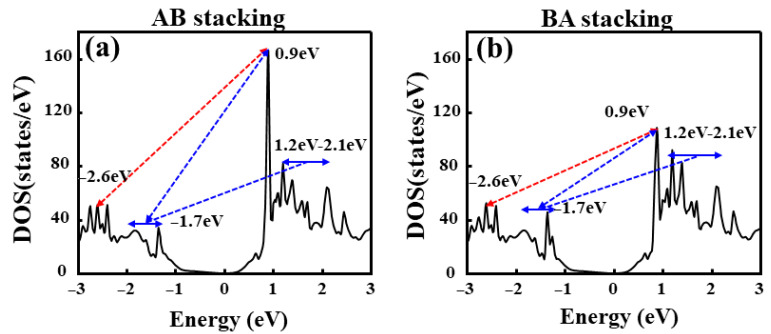
DOS based on the S–S configuration: (**a**) for the AB-stacked configuration and (**b**) for the BA-stacked configuration.

**Figure 11:**
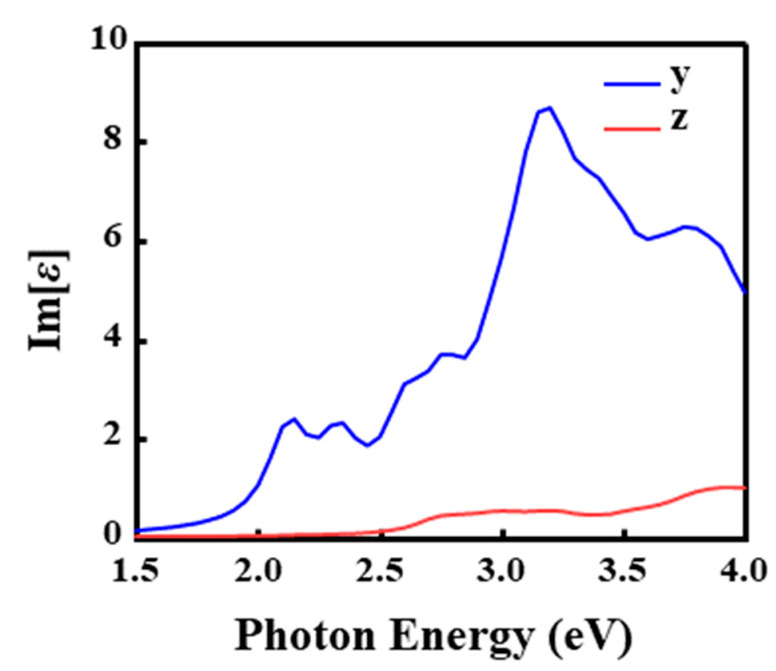
Dielectric constants in the S–S configuration along the y and z-directions.

To understand the physical origin of this anisotropy beyond phenomenological description, we analyze the density of states (DOS, Figure 10) and the complex dielectric function (Figure 11). The DOS analysis provides a clear explanation for the peak positions. According to Fermi’s golden rule, the probability of electron transitions from the valence band to the conduction band is closely related to the DOS of the initial and final states. Therefore, the main peaks in the photocurrent often show good energy alignment with the DOS peaks of the valence and conduction bands. The DOS analysis provides a clear explanation for the peak positions. For the AB stacking, distinct DOS peaks are observed at −2.6 eV (valence band) and +0.9 eV (conduction band), yielding an energy difference of 3.5 eV that perfectly matches the z-polarized photocurrent peak. Furthermore, a shoulder peak appears at −1.7 eV, creating a gap of 2.6 eV with the conduction band peak, which aligns precisely with the redshifted y-polarized photocurrent peak.

However, while DOS alignment explains the energy resonance, it does not account for the intensity disparity (y > z). This anisotropy originates from the quantum mechanical orbital selection rules. In monolayer and bilayer TMDs, the electronic states at the band edges are predominantly composed of Mo d-orbitals (e.g., d_z2_, d_xy_, d_x2-y2_) [73]. For in-plane polarized light, the electric field vector couples efficiently with the planar spread of the d-orbitals, satisfying the symmetry requirements for allowed transitions. Conversely, for out-of-plane polarized light, many transitions near the band edge are symmetry-forbidden or strongly suppressed due to the orthogonality of the orbital wavefunctions. This mechanism is directly corroborated by the imaginary part of the dielectric function, Im[*ε*] (Figure 11), where Im[*ε*]_y is significantly larger than Im[*ε*]_z in the 2.0–3.5 eV range. Thus, the bilayer Janus MoSSe homojunction functions not only as a switchable photodetector but also as a polarization-sensitive device governed by intrinsic quantum rules.

**Figure 12:**
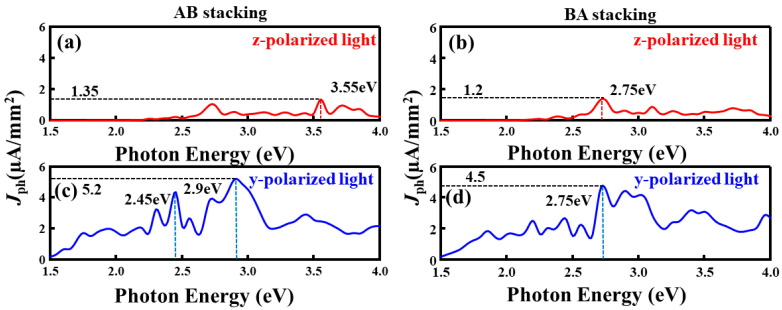
Photocurrent device based on the Se–Se configuration: (**a**) photocurrent response of the AB stacking under z-polarized illumination; (**b**) photocurrent response of the BA stacking under z-polarized illumination; (**c**) photocurrent response of the AB stacking under y-polarized illumination; (**d**) photocurrent response of the BA stacking under y-polarized illumination.

Having established the microscopic origin of the stacking-dependent and polarization-sensitive photoresponse in the S–S configuration, we further examine whether these trends remain valid for other bilayer MoSSe stacking types. As shown in Figure 12, the Se–Se configuration exhibits the same qualitative behavior: the AB stacking consistently produces a higher photocurrent than the BA stacking, and the in-plane (y-polarized) illumination leads to a markedly stronger response than the out-of-plane (z-polarized) illumination. This confirms that the anisotropic and ferroelectric-tunable photoresponse is a robust feature of bilayer Janus MoSSe devices rather than a special case of the S–S configuration.To further verify the generality of the above trends, we examine the Se–Se and S–Se configurations. The Se–Se photocurrent spectra are shown in Figure 12, while the corresponding DOS and dielectric analyses (Figures S3 and S4) and the full set of results for the S–Se configuration (Figures S5–S7) are provided in the Supplementary Materials. For the Se–Se configuration, when the light polarization is along the z direction (Figure 12a,b), the main photocurrent peaks of the AB and BA stackings are located at approximately 3.55 eV and 2.75 eV, respectively, and the peak photocurrent intensity of the AB stacking is clearly higher than that of the BA stacking. When the light polarization is switched to the y direction (Figure 12c,d), the main photocurrent peaks of both stackings shift overall toward lower energies. The AB stacking exhibits two pronounced peaks at 2.45 eV and 2.9 eV, whereas the BA stacking shows only a single dominant peak near 2.75 eV. Moreover, the photocurrent intensity under y-polarized light is generally higher than that under z-polarized light. The corresponding DOS distribution (Figure S3) and the imaginary part spectra of the dielectric constant (Figure S4), further indicate that the AB stacking exhibits a higher DOS peak in the relevant energy range and a greater dielectric constant along the y-direction, which are the fundamental reasons for its stronger photocurrent. The photocurrent, DOS, and dielectric-function results for the S–Se configuration (Figures S5–S7) exhibit trends similar to those of the other two configurations. Specifically, the combination of AB stacking and y-polarized light yields the best photoresponse, featuring higher photocurrent intensity and more pronounced anisotropy. To comprehensively evaluate device efficiency, photoresponsivity *R*_ph_ is also crucial. In the Supplementary Materials (Figures S8–S10), we provided the calculation method for the photocurrent responsivity *R*_ph_ and the corresponding results.

## 4. Conclusions

In this work, we have systematically investigated the ferroelectric switching, quantum transport, and optoelectronic properties of bilayer Janus MoSSe lateral homojunctions using first-principles calculations combined with the NEGF method. For electronic applications, we constructed a 1T/2H/1T lateral homojunction, which successfully reduces the contact barrier and minimize interface scattering. In this pristine electrostatic environment, the sliding-induced polarization reversal triggers a drastic reconstruction of the local density of states and the effective tunneling barrier in the 2H channel, leading to a giant TER up to the order of 10^4^%. This represents a performance improvement of several orders of magnitude compared to traditional vertical junctions. In parallel, for optoelectronic applications, we explored a lateral H-phase p-i-n junction. We found that the photocurrent response is highly tunable by both the ferroelectric stacking order (with the AB state outperforming the BA state) and the light polarization direction. Specifically, the device exhibits strong optical anisotropy, where the in-plane (y-polarized) response is significantly enhanced due to the orbital selection rules of Mo-d states. The superior performance of the AB stacking over the BA state is attributed to its more favorable DOS distribution, which exhibits higher DOS peaks at the relevant excitation energies, thereby enhancing interband transition probabilities and photocurrent generation. Overall, this work establishes a clear correlation between polarization state, local electronic structure, and transport/optoelectronic performance, providing a solid theoretical foundation for the design of high-performance non-volatile memories and reconfigurable two-dimensional optoelectronic devices.

## Figures and Tables

**Table 1:** Comparison of TER Values in Different Studies.

Reference	Channel Material	Electrode	Device Geometry	TER (%)
[39]	SnSe	Doping	lateral	1.46 × 10^3^
[61]	h-BN/In2Se3(monolayer)	Au	vertical	170
[61]	h-BN/In2Se3(bilayer)	Au	vertical	1193
[62]	MoSSe	Au	vertical	263–313
[63]	SnS	Doping	lateral	2600
This study	2H-MoSSe	1T-MoSSe	lateral	5.3 × 10^4^

## Data Availability

The data presented in this study are available on request from the corresponding author.

## Supplementary Materials

The following supporting information can be downloaded at: https://www.mdpi.com/article/10.3390/nano16060370/s1. Figure S1: Schematic illustration of the transition between two FE phases of the Se–Se configuration; Figure S2: Schematic illustration of the transition between two FE phases of the S–Se configuration; Figure S3: DOS based on the S–Se configuration; Figure S4: Dielectric constants in the Se–Se configuration; Figure S5: Photocurrent Response of Devices Based on S–Se Structures; Figure S6: DOS based on the S–Se configuration; Figure S7: Dielectric constants in the S–Se configuration; Figure S8: Photocurrent device based on the S–S configuration: (a) Responsivity of the AB stacking under z-polarized illumination; (b) Responsivity of the BA stacking under z-polarized illumination; (c) Responsivity of the AB stacking under y-polarized illumination; (d) Responsivity of the BA stacking under y-polarized illumination; Figure S9: Photocurrent device based on the Se–Se configuration: (a) Responsivity of the AB stacking under z-polarized illumination; (b) Responsivity of the BA stacking under z-polarized illumination; (c) Responsivity of the AB stacking under y-polarized illumination; (d) Responsivity of the BA stacking under y-polarized illumination; Figure S10: Photocurrent device based on the S–Se configuration: (a) Responsivity of the AB stacking under z-polarized illumination; (b) Responsivity of the BA stacking under z-polarized illumination; (c) Responsivity of the AB stacking under y-polarized illumination; (d) Responsivity of the BA stacking under y-polarized illumination.
