# Supplementary material for: Giant Tunneling Electroresistance and Anisotropic Photoresponse in Sliding Ferroelectric Homojunctions Based on Bilayer Janus MoSSe
Source: Nanomaterials (Basel). 2026 Mar 18;16(6):370. doi: 10.3390/nano16060370 (PMC13029547; doi:10.3390/nano16060370)
Supplement: Supplementary file 1 [file nanomaterials-16-00370-s001.zip › nanomaterials-4141298-supplementary.pdf]

Supplementary Materials

This Supplementary Materials document provides additional datasets and analyses that support the main conclusions of the manuscript, while keeping the main text concise and focused. In particular, **Figures S1–S2** provide further evidence for the low-barrier interlayer-sliding ferroelectric switching in alternative stacking types, and **Figures S3–S7** provide complementary electronic-structure and optical-response analyses that rationalize the stacking- and polarization-dependent photocurrent behaviors discussed in Section 3.3, and Figures S8–S10 present the corresponding responsivity spectra for different stacking configurations.

S1-S2: Additional verification of sliding ferroelectric switching in Se–Se and S–Se configurations

In the main text (Section 3.1), we demonstrate that bilayer Janus MoSSe exhibits interlayer-sliding ferroelectricity, where AB and BA stackings form two switchable polarization states separated by a small kinetic barrier. To further verify that this low-barrier switching is not limited to the S–S stacking, we provide the corresponding switching pathways for the Se–Se and S–Se configurations in Figures S1 and S2, respectively. The consistently small energy barriers confirm that sliding-mediated polarization reversal is energetically accessible across different stacking types.

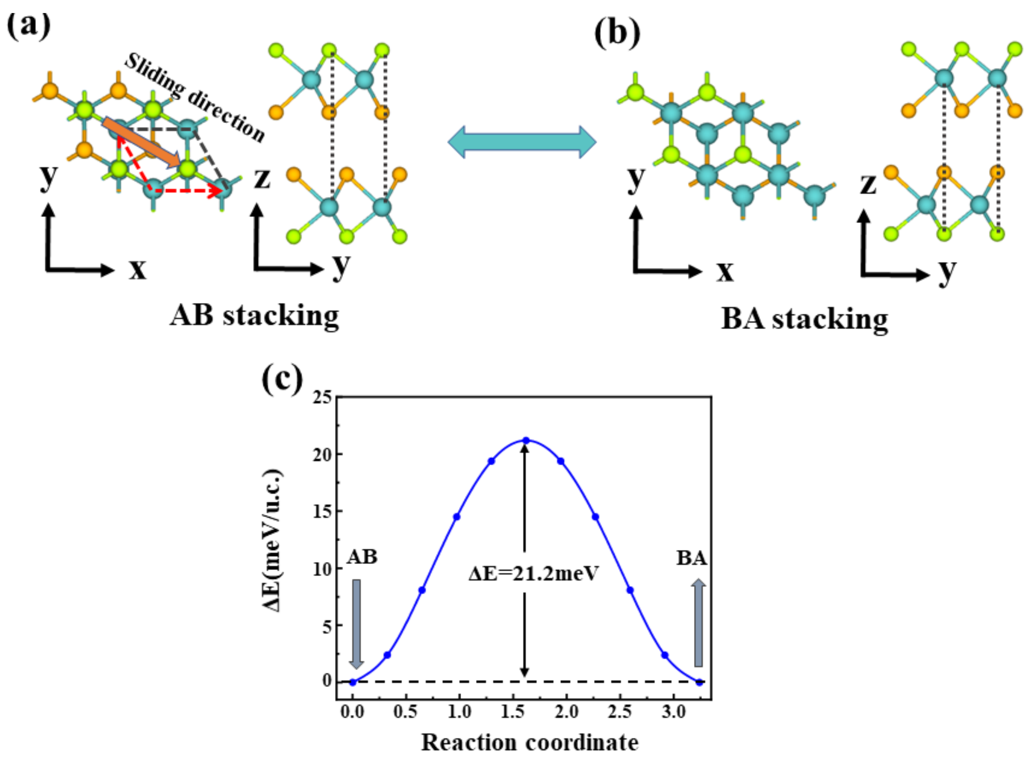

Figure S1. Schematic illustration of the transition between two FE phases of the Se–Se configuration: (**a**) top and side views of the AB-stacked structure; (**b**) top and side views of the BA-stacked structure; (**c**) The transition energy barrier along the ferroelectric switching pathway.

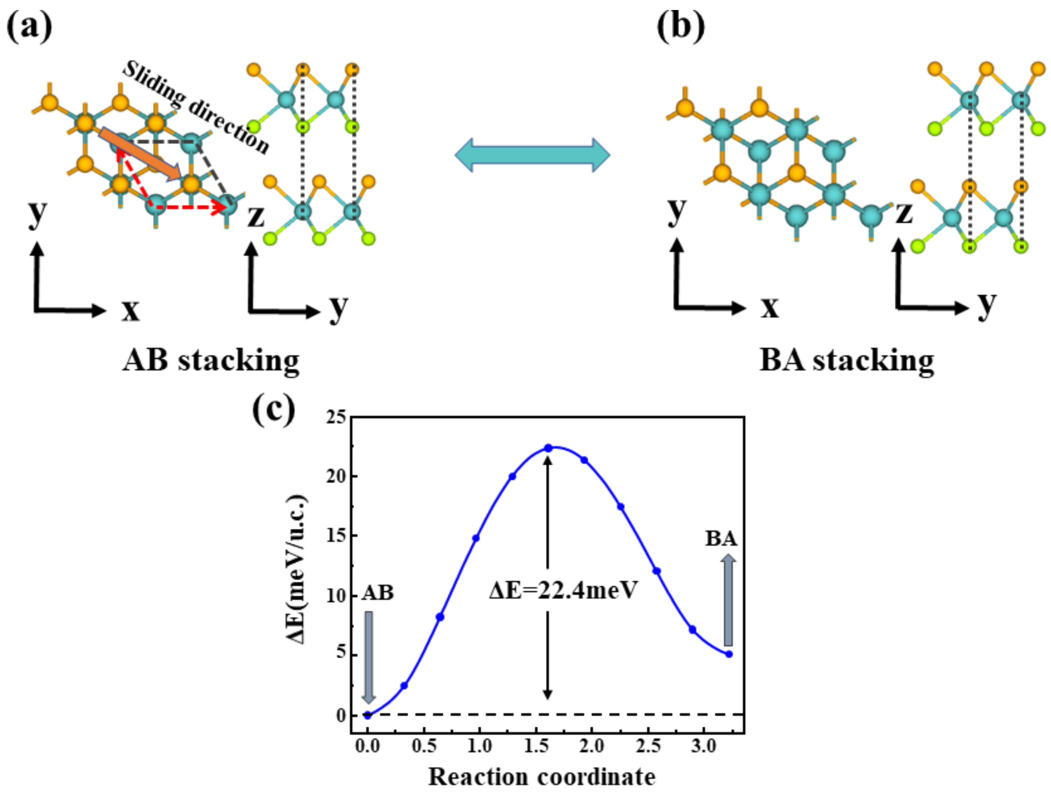

Figure S2. Schematic illustration of the transition between two FE phases of the S–Se configuration: (**a**) top and side views of the AB-stacked structure; (**b**) top and side views of the BA-stacked structure; (**c**) The transition energy barrier along the ferroelectric switching pathway.

S3-S7: Supporting electronic-structure and optical-response analyses for photocurrent anisotropy

In Section 3.3 of the main text, we show that the p–i–n photodetector based on bilayer MoSSe exhibits pronounced optical anisotropy, with stronger in-plane (y-polarized) photoresponse than out-of-plane (z-polarized) response, and with the AB stacking generally outperforming BA. To provide microscopic understanding and additional validation for other stacking types, **Figures S3–S7** present the density of states (DOS) and dielectric-function analyses for the Se–Se and S–Se configurations, together with the corresponding photocurrent spectra for the S–Se configuration. In addition, Figures S8–S10 show the corresponding responsivity spectra for the S–S, Se–Se, and S–Se configurations.   Together, these results support the interpretation that (1) the dominant photocurrent peak positions are closely related to DOS features in the valence and conduction bands, and (2) the stronger in-plane response originates from intrinsic orbital-selection rules and enhanced optical transition strength along the in-plane direction.

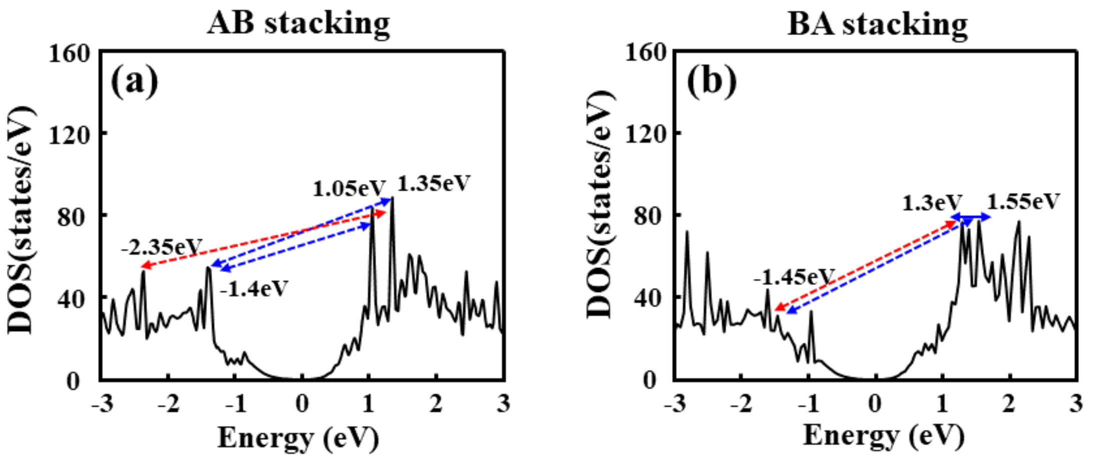

Figure S3. DOS based on the Se–Se configuration: (**a**) for the AB-stacked configuration and (**b**) for the BA-stacked configuration.

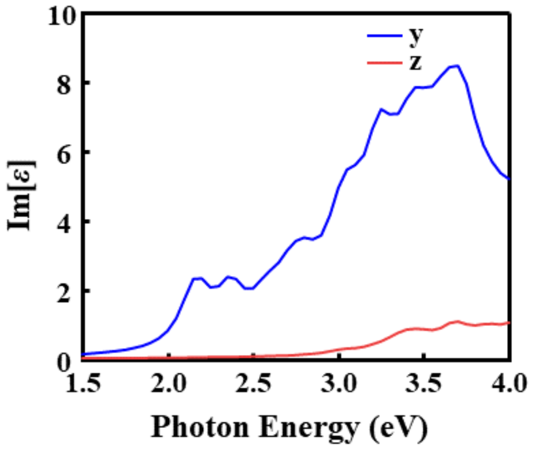

Figure S4. Dielectric constants in the Se–Se configuration along the y and z-directions.

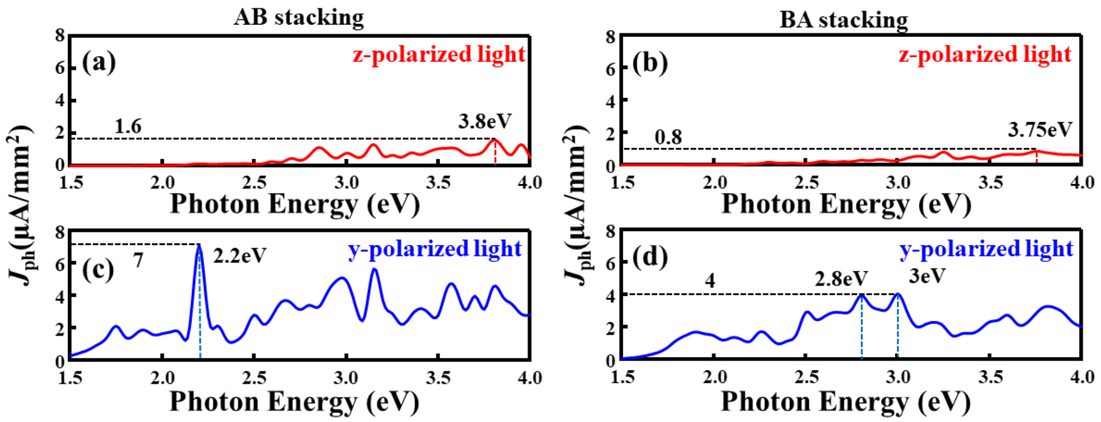

Figure S5. Photocurrent device based on the S–Se configuration: (**a**) photocurrent response of the AB stacking under z-polarized illumination; (**b**) photocurrent response of the BA stacking under z-polarized illumination; (**c**) photocurrent response of the AB stacking under y-polarized illumination; (**d**) photocurrent response of the BA stacking under y-polarized illumination.

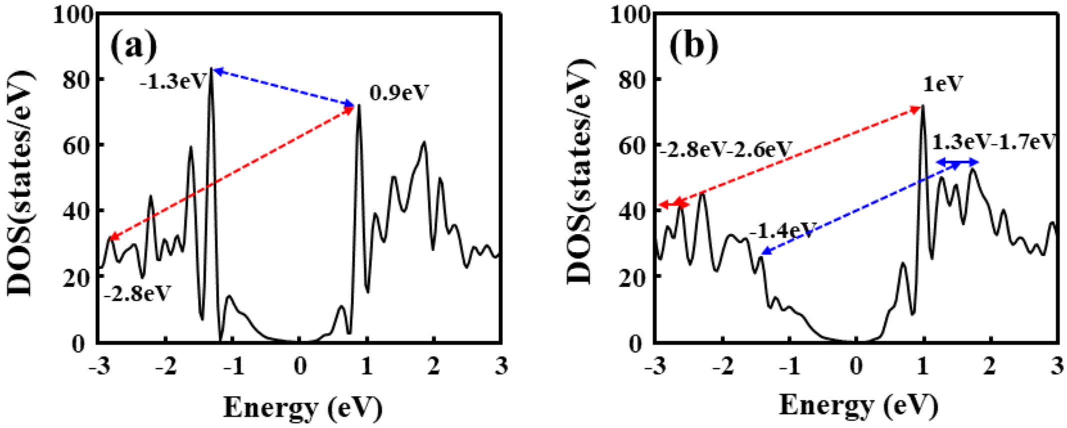

Figure S6. DOS based on the S–Se configuration: (**a**) for the AB-stacked configuration and (**b**) for the BA-stacked configuration.

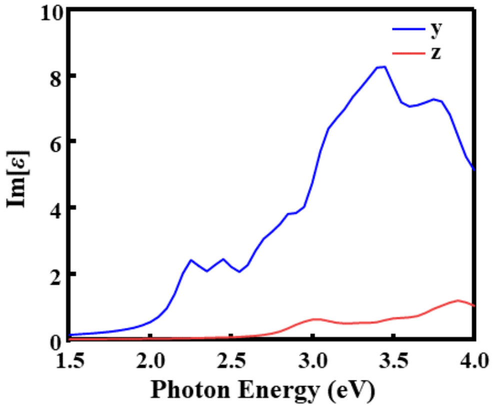

Figure S7. Dielectric constants in the S–Se configuration along the y and z-directions.

Furthermore, we investigated the Responsivity R_{ph} (can be defined from our simulations). Responsivity is defined as the photocurrent generated per incident optical power,

$$R_{ph} = \frac{J_{ph}}{I_{\omega}E}$$

where E is the photon energy, I_{ω} is the photon flux. The calculated responsivity spectra for the S–S, Se–Se, and S–Se configurations are presented in Figures S8–S10, respectively.

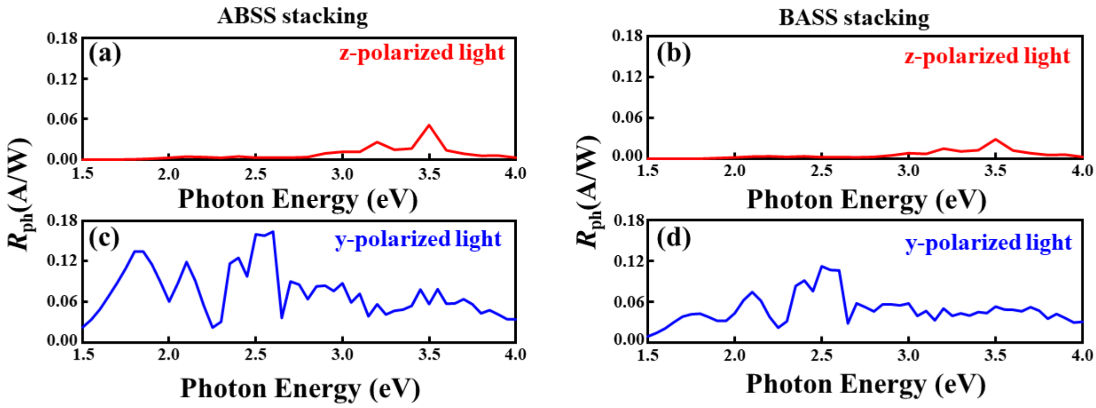

Figure S8. Photocurrent device based on the S–S configuration: (**a**) Responsivity of the AB stacking under z-polarized illumination; (**b**) Responsivity of the BA stacking under z-polarized illumination; (**c**) Responsivity of the AB stacking under y-polarized illumination; (**d**) Responsivity of the BA stacking under y-polarized illumination.

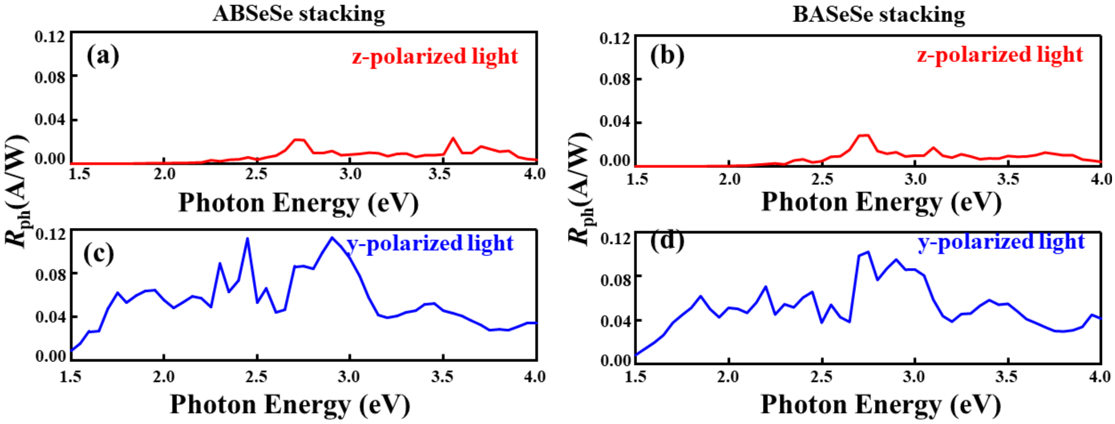

Figure S9. Photocurrent device based on the Se–Se configuration: (**a**) Responsivity of the AB stacking under z-polarized illumination; (**b**) Responsivity of the BA stacking under z-polarized illumination; (**c**) Responsivity of the AB stacking under y-polarized illumination; (**d**) Responsivity of the BA stacking under y-polarized illumination.

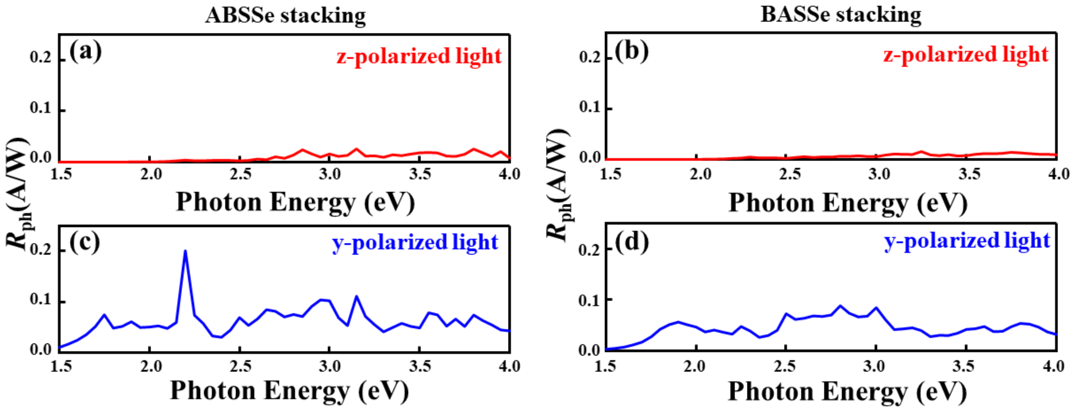

Figure S10. Photocurrent device based on the S–Se configuration: (**a**) Responsivity of the AB stacking under z-polarized illumination; (**b**) Responsivity of the BA stacking under z-polarized illumination; (**c**) Responsivity of the AB stacking under y-polarized illumination; (**d**) Responsivity of the BA stacking under y-polarized illumination.
